# The pivotal role of open source knowledge transfer to achieve universal energy access

**DOI:** 10.1016/j.isci.2025.112093

**Published:** 2025-02-28

**Authors:** Sanli Faez, Vivien Barnier, Dimitrios Mentis

**Affiliations:** 1 Debye Institute for Nanomaterials Science, Princetonplein 1, Utrecht, the Netherlands; 2 EnAccess Foundation, Industrieweg 9, Voorschoten, the Netherlands; 3 World Resource Institute, 10 G Street NE, Suite 800, Washington, DC 20002, USA

**Keywords:** Energy policy, Open source software, Energy sustainability, Energy systems

## Abstract

Community-company collaboration based on open source technologies is emerging as an alternative to proprietary solutions for planning, implementation, and maintenance of localized access to clean renewable electricity. We highlight some recent breakthroughs in creation of local technology ecosystems that follow an open access approach to knowledge transfer. On the other hand, on top of engineering, manufacturing, and maintenance questions, community led projects need to address social acceptance, economic viability, and regulatory barriers. Despite these barriers, open source in energy access is expanding and new sustainable business models are practiced. By comparing the advances in open source hardware scholarship with that of free and open source software, we anticipate an increasing communal pressure for adopting open-source friendly innovation policies. Academia and in particular university knowledge transfer policies play an essential role in achieving universal energy access via open source technologies.

## Graphical abstract

**Figure:**
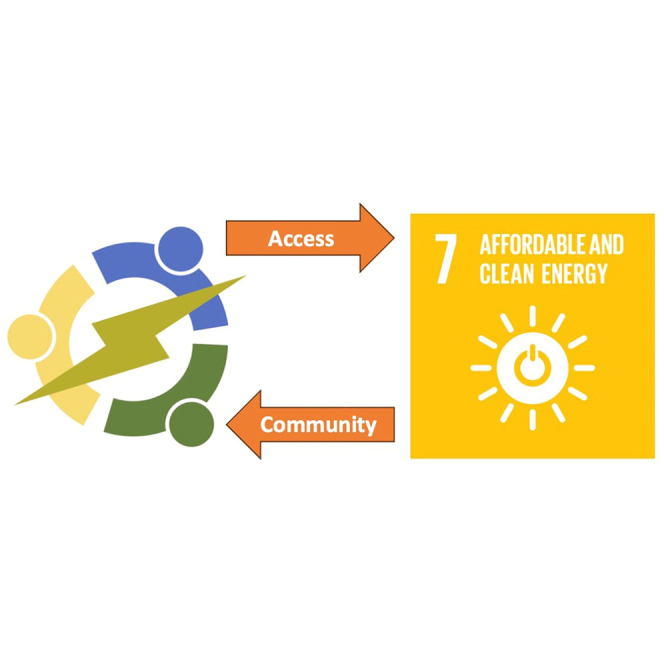

## Introduction

**Figure 1:**
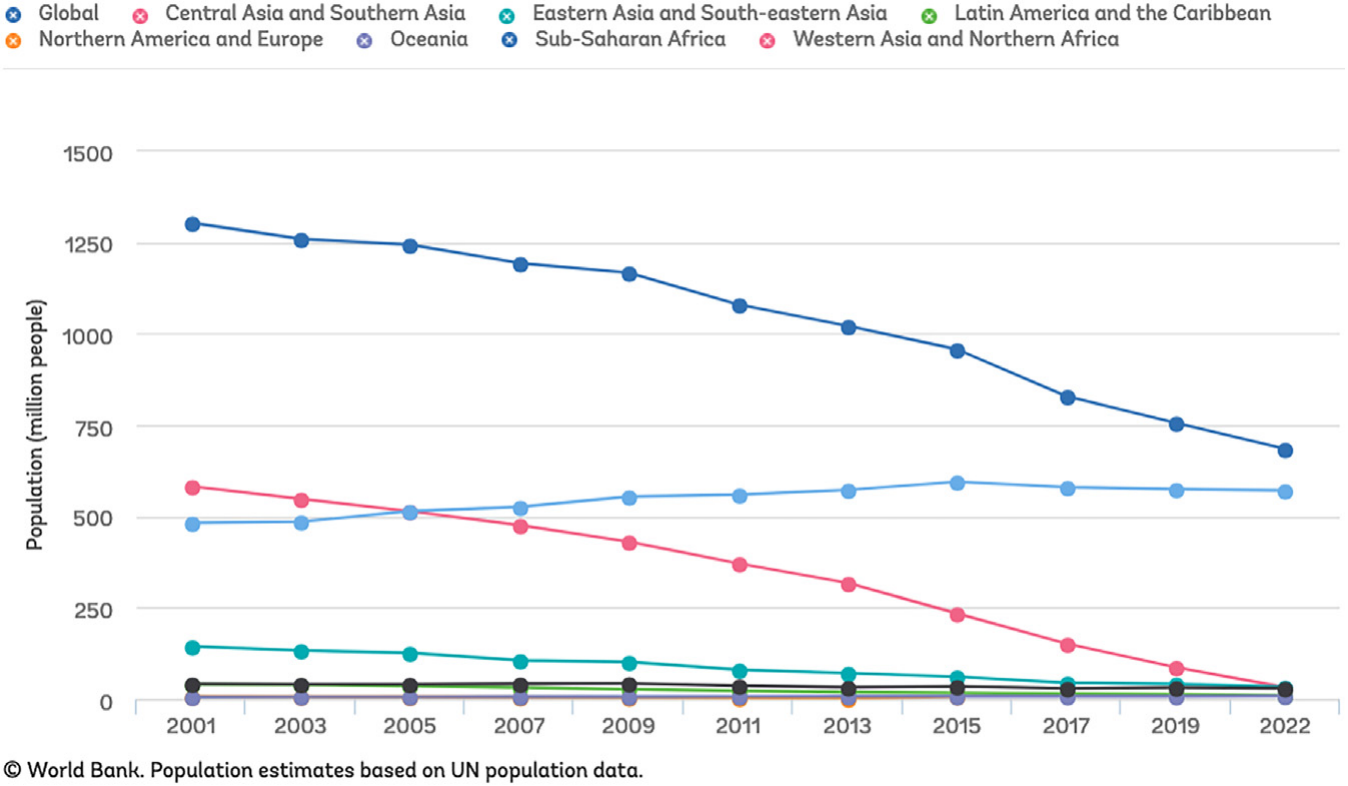
Annual trends of population with no access to electricity An increasing population in sub-Saharan Africa have no access to electricity, against the global trend and the goals set by United Nations SDG7.

Worldwide, close to 700 million people have no access to electricity and over 2 billion people have no possibility of cooking with clean fuels and technologies (source: World Bank, 2022, https://trackingsdg7.esmap.org/). United Nations Sustainable Development Goal 7 has aimed at “access to affordable, reliable, sustainable and modern energy for all” by 2030. Nine years after setting this goal, there has been a remarkable progress in providing energy in the Global North and most parts of Asia, but the progress is worryingly falling behind in sub-Saharan Africa (see Figure 1). Considering the urgency of reducing dependence on fossil fuels, accelerating access to clean energy sources requires innovative approaches to planning, production, and maintenance.

One of these radically distinct approaches to accelerating access to renewable energy sources is to release the immense potential of collaborative models and creating open source technology ecosystems. In this perspective, we highlight the advantages and challenges of taking the open source route to energy access. We study the case of Open Source Energy Access Symposium (OSEAS) in Abuja as an exemplary forum where some of these advantages and challenges are discussed.

Empirical research has demonstrated how open source development has increased innovation and technology adoption in middle- and high-income economies while reducing costs across various domains.^1^^,^^2^^,^^3^ The most visible universal impact of open source technologies is in the informatics sector. As a relatively mature technology, free and open source software (FOSS) is the most studied open source ecosystem in the scholarly literature. A recent study on the role of open source software in the energy sector has collected and analyzed hundreds of open source projects in this sector.^4^ This study has concluded that open source software has been successful in transforming industries by accelerating development, lowering costs, and ensuring stability and security. The majority of projects have been initiated or are currently driven by academic contributors. Despite that, the authors conclude that the commercial energy sector has benefited from applying open source principles to address the unique challenges for expanding the renewable energy network. The collaboration between academic and commercial players in open source projects creates mutual benefits particularly by standardization,^5^ increasing access to data, and interoperability of services across the value chain.

**Figure 2:**
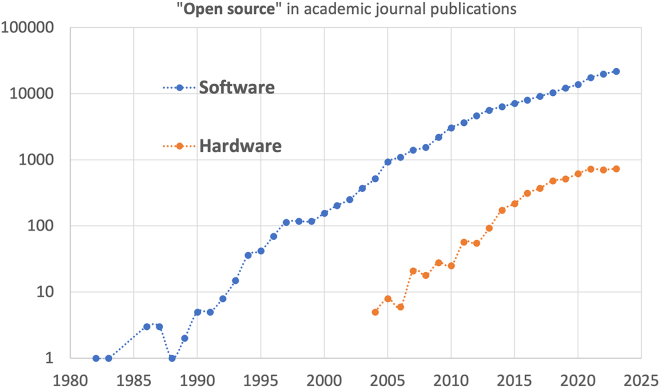
Publication trends for open source software and hardware Number of scientific publications mentioning “open source software” and “open source hardware” in their title or abstract plotted against the publication year. Parallel trends over almost two decades indicate to a similar scaling with a delay of about 15 years. Source: Scopus.

After half a century of developing FOSS, despite the initially fierce resistance of the big software corporation, open source software has become one the main pillars of computing and information technologies. An overall positive impact on economic growth has been measured even for high-income economies.^6^^,^^7^ Looking at the trend of scientific publications related to open source software and open source hardware in Figure 2, it is obvious that the latter is trailing the expansion of the former with a delay of about 15 years. With the expansion of the open science movement worldwide, we expect that this increasing trend will continue, or even accelerate. Therefore, one can anticipate that in year 2040, open source hardware will play a proportionally prominent role as open source software is doing today. Considering the rapid adoption of open source technologies by local small businesses active for the energy sector in majority countries, we expect this transition to be driven from local initiatives, forcing the big corporation to follow course. Open source planning software and generic interoperable hardware (not strictly open source) are already playing a central role in a growing ecosystem of small and medium energy providers deploying minigrid systems in under-served communities. These minigrid electricity suppliers play a crucial role in the clean energy transition by providing access to African communities in areas that investing in large grids is lagging behind. Unlike big infrastructure projects, minigrids are flexible, dependable, and financially feasible for small and medium enterprises (SMEs) to set up and maintain.^4^ Under effective planning conditions, the deployment of renewable energy sources through minigrid systems may be considered a more viable approach to supplying productive electricity to rural communities.^8^^,^^9^^,^^10^ This development is akin to the leap from no telephone access to mobile communications in many locations in Africa, skipping the land-line networks completely. While SMEs can play a crucial role in expanding and sustaining the renewable energy access in African nations they cannot invest in major upstream resources and tools such as data-driven planning services or development of key basic technologies such as batteries or power electronics^11^^,^^12^ or financial transaction systems for commercial viability of these energy suppliers.^13^ These are some examples of areas that still require global collaboration and major investment in research and development of open source technologies. The open source development of these technologies is a fertile ground for fair and inclusive collaborations. A bi-directional knowledge flow between technology and service providers and user communities is inherently necessary as these tools can only be deployed properly when reliable data on energy needs, actual demographics, and potential use-cases are made available from the local operators.

## Discussion

### Case study: Open source planning tools

Especially in sub-Saharan Africa, the demand for reliable electricity is still surpassing supply. It remains a challenge to thoroughly assess the energy consumption needs of communities that are still not even electrified. To fulfill the needs of external investors, local operators should demonstrably identify communities with sufficient resources and economic growth potential to secure a viable and durable impact of the investment. On the national or subnational scale, this exploration is mainly performed using geospatial planning tools that use a combination of data sources to estimate the optimal distribution of resources and identify potential risks. Developing such a complex platform is far beyond the capacity of a single SME. Hence, there is a large demand for affordable geospatial planning solutions that lower the entry barrier to the energy-providing sector. To answer this need the Energy Access Explorer (EAE) was initiated by the World Resource Institute,^14^ which was the first fully open source and locally led planning tool. The initial aim of the project at its conceptualization was to reach 15 institutional users by year 2020. Based on usage tracking, the beneficiaries of the EAE are now exceeding 25,000 users, about fifteen hundred times larger than the initial aim. The majority of these users are small local businesses. This outstanding reach, beyond the initial expectations, highlights the opportunities that arise from making such platform open source. Both the service providers as well as for the local operators benefit from the open access. Meanwhile, there are several other solution providers that are also walking the open access route; see Table 1 for non-exhaustive list. While expansion of the open source toolbox is a generally positive trend, parallel development of distinct and not inter-operable open source tools have some negative sides that we will discuss later in the section “pitfalls of open source development.”

**Table 1:** A list of open source software tools for geospatial planning

OpenTEPES	Open-access tool for energy planning, providing full functionality for determining investment plans for new power facilities.	^15^
OSeMOSYS	Energy modeling tool that has been developed to support energy planning processes, offering transparent and freely available energy modeling practices.	^16^
Optihood	Software framework that allows for multi-objective optimization and analysis of energy systems for neighborhoods.	^17^
Autarky	Software for stochastic optimization of grid-connected mini-grids, accounting for uncertainties, like main grid blackouts.	^18^
COIN-OR	Linear programming (CLP) solver for computing solutions for large-scale electricity system planning models.	^19^
OnSSET	Open Source Spatial Electrification Tool—estimates technology and investment requirements to reach electrification targets.	^20^

### From open source planning to open source distributed manufacturing

Next to effective planning based on digital tools and data, access to clean electricity requires materials and physical equipment. Fortunately, in contrast with high-CapEx infrastructure projects, mini-grids are inherently compatible with distributed manufacturing. Many solution providers in low coverage areas are small and medium businesses (SMEs). In the relatively young renewable energy market, hardware manufacturers are still diverse in scale and quality. It can thus be a major overhead cost for these SMEs to evaluate and build durable supply chains. Therefore, the minigrid supply chain and maintenance ecosystem can still benefit a lot from standardization and collaboration, to collectively reach an economy of scale. Some pioneers have created open source hardware technologies for various elements of a minigrid such as for monitoring,^21^ electric vehicle (EV) charging,^22^ battery management,^23^ or design of the complete system.^24^

In a growing market where demand far exceeds supply, protection of intellectual property for the sake of vendor lock-in is a general hindrance with a high financial and opportunity cost, and little or no benefit on productivity.^25^ Relying on specialized non-standard parts that are not at the core business of each company reduces the overall impact of the sector in comparison with incumbent fossil-based energy providers. Particularly, in a sector with a significant share of public and philanthropic money being used, focus on maximizing impact and reach should get a higher priority than market domination. Based on this realization, some of the philanthropic investors have focused on de-risking the development of open source technologies by directly financing those to keep the source open.

### Case study: Investing exclusively on open source

One example of such philanthropic initiative is the EnAccess foundation. Since 2007, this foundation has focused on removing the barriers to knowledge sharing at the system level. One of the most successful examples of EnAccess’s open source policy is the OpenPayGO Token, which is compatible across different platforms, and manufacturers.^13^^,^^26^ Pay As You Go (PAYG) is a business model that has gained considerable traction by breaking down purchases of equipment and fuel into affordable micropayments and providing a mechanism through which low-income households can access new technologies. The pure encryption and decryption of access tokens is generally of no business value to any of those actors, but the lack of orchestration had created incompatible codes and unnecessary complexity. The OpenPayGO Token has pushed several other operators in Africa to support interoperability and hence become more efficient.

Serving the same community of stakeholders, EnAccess and World Resources Institute (WRI) jointly organized the first OSEAS 2024. A very diverse audience, from open source developers and academic researchers to government officials, donor representatives, and business owners were brought together in Abuja, Nigeria, to share their insights and co-design a path for stronger collaboration in the future. The conscious choice of organizing the symposium in Nigeria had enabled strong presence from countries in the global south.

This growing community is undoubtedly unified on the higher goal of making universal access to clean sustainable energy a reality. By sharing the overhead costs of technology development, project planning, and education, these actors have achieved a great deal in electrifying regions that had insufficient access to modern energy services. The diversity of the speakers in gender, background, and specialization was remarkably broad. This need for diversity, both in the open source developers community, and energy access was emphasized repeatedly during the symposium.

The organizers highlight these 10 recommendations in their final report.

(1) Leverage open-source platforms like the Global Electrification Platform for cost-effective, standardized geo-spatial analysis in electrification planning.
(2) Develop training programs to build capacity among energy professionals in developing countries for using open-source tools in energy planning.
(3) Form partnerships with universities, research organizations, and stakeholders to develop and implement open-source solutions and replicate successful models.
(4) Engage end-users, such as health and education ministries, in planning to ensure solutions align with local needs and priorities.
(5) Ensure open-source tools are transparent, publicly available, and user-friendly, with adequate training and support.
(6) Increase funding for research and development in high-capacity, affordable battery technologies, focusing on both fundamental research and applied innovation.
(7) Foster international collaboration for technology transfer, best practices sharing, and learning from global renewable energy projects to speed up the energy transition.
(8) Enhance women’s participation in technology design, testing, and maintenance, involving them as active contributors.
(9) Invest in capacity-building to raise awareness of funding sources and improve data management for reliable investor engagement.
(10) Define roles and responsibilities in data governance and promote collaboration among stakeholders for effective data management.

These recommendations highlight the continuing ambitions of the open source community to achieve tangible progress in the energy sector. It is also an open call to international organizations and policy makers to become more conscious of the benefits of using the public domain innovations and invest in the infrastructure that can support this energy access commons.

### Pitfalls of open source development

While open source projects are praised for lowering the entry barrier for contributors, as bottom up initiative they often lack a comprehensive governance structure or a resources development roadmap. Some projects thin down by branching into parallel track or turn into niche applications that are not all interoperable. Although, existence of alternatives and avoiding a monopoly is generally seen as a positive factor that ensures innovation and improves the services, every extra planning tool offered will cause extra overhead to adopters such as the local and national government for assessment and selection. The lack of coordination in providing planning tools is inefficient because of these assessment costs that can cause delays in the implementation. A collaborative approach to quality control and task distribution between the various open source providers can create a good common ground for ensuring an adequate and efficient high quality service and preservation of community values.

For the case of geo-spatial planning services the OSEAS symposium served as an opportunity for collaboration between so far distinct open source planning projects. The project representatives shared their vision and decided to develop a common roadmap for collaboration in the upcoming year and to present that in the next symposium.

### Market-driven open source business models

Beside advantages for resource sharing, innovation acceleration, and standardization, the recent path of adopting open source software and hardware by some of the biggest tech-companies has proven that adopting open source can be deliberate and rational economic choice. Some companies emphasize their choice of open source to emphasize transparency and reliability of their products, as a market advantage, or to rapidly obtain a larger user base. Dedicating resources of (for-profit) companies to maintaining open source project is justified based on the cost savings for reliability, ease of maintenance, product compatibility, and sustaining upstream dependencies.

Next to well-established open-source projects that benefit from the critical mass of their developer communities, some start-ups choose to build their business around these established or emerging open source technologies based on new and creative business models.^27^^,^^28^

These models include, but are not limited to the following:

(1) The open core model: The core of the software or hardware is open source, but additional proprietary features or enhancements are offered for a fee.
(2) Services around open source products: Commercial services are offered around free and open source products; for example consulting, training, and technical support. This model uses specific expertise to generate revenue without earning from selling the product itself.
(3) Platform/data service providers: Under this model, businesses provide hosting of open-source products and rent the service to users via subscriptions or customized services. This model adds value for users through ease of use, scalability, and maintenance, while the provider can benefit from the economy of scale.
(4) Branding and reselling: For some software products and especially for open-source hardware, end users are willing to pay an additional fee for quality management and after-sale services. This model has been successfully applied to some consumer-grade open source hardware.

Also, a combination of different models is being used by companies. For instance, providing paid premium hosting services and offering additional services around the products work greatly together.

### On the role of academia

While the local governments, businesses, civil society, finance institutions are focusing on extending services to access-deprived regions with the existing tools and technologies, medium-long term sustainability of open source solutions remains a challenge. This is an area where academic institutions can contribute through curriculum development, training of trainers, and adopting and enhancing open source technology as part of academic dissertations.^1^^,^^29^

When focusing on distributed manufacturing and services, long-term maintenance is almost as important a design factor as innovation in performance. While academic research is often focused on innovation and pushing the boundaries for production or storage efficiency, without maintenance, such solutions will be of little use for the long-term use by local service providers and end-users. Therefore, it is important to consider the availability of local resources and expertise as part of the technical solutions. Open source solutions are transparent and inherently extendable to the complete material and technological chain of supply. Additionally, the usefulness of any technological solution is ultimately determined by its fitness to local conditions and constraints. The flexibility of open source solutions and involvement of the local developers community accommodates matching of needs and services from the bottom up, including the local higher education providers. The reduced cost of creative commons licensing removes the financial barrier for low-income countries in using proprietary solutions.

For a faster scaling of such capacities and a natural embedding of the regional ecosystem of knowledge, it is commendable for well-established academic groups to invest in partnership with local academic institutions. This route requires a long-term perspective on knowledge transfer, but can offer a more sustainable and rapidly scaling up by domesticating the necessary expertise and adaptation to local geopolitical and human capacities. Local academic institutions can play a crucial role in developing a skilled workforce for the energy sector, particularly in the fields of high social relevance. Furthermore, such collaboration can lead to the alignment of policy recommendations for international donor agencies, national policymakers, and local development planners, thereby addressing existing challenges and future prospects of preserving the locally available expertise.

### The path forward

The open science movement has grown from a progressive effort focusing on best scientific practices to the mainstream of funding policies. The United States federal government announced 2023 as the year of open science featuring several national initiatives. In the same year, United Nation’s United Nations Educational Scientific and Cultural Organization (UNESCO) published its recommendations on open science as a framework for making knowledge inclusive, equitable, and sustainable. It is one of the recommendation of the UNESCO^30^ open science policy paper that well-established research universities of the global north can further enhance the open source knowledge transfer by adopting a more permissive and non-exclusive licensing regime for their innovations that are at lower technological readiness level. The UNESCO recommendations also recommend working with open principles to accelerate toward reaching the sustainable development goals. Wide spread adoption of this policy can be a game changer in democratizing and enriching access to clean technologies, in the global north and global south simultaneously.

This mindset shift requires a higher awareness of the benefits and impact of open source solutions among the governance boards and especially the knowledge transfer offices of established research universities.^2^^,^^31^

Meanwhile, bottom up initiative can become more coherent by forming open source communities around open source project. The high moral motivation for accelerating the transition to renewable energies, to overcome the worse scenarios of climate, is a unifying element that attracts the new generation of researchers and energy practitioner to choose open source innovations for contributing to the common good.

### Limitations of the study

This perspective article is mainly built on the experience of the authors with development of open source solutions over the past ten years and their interaction with the expanding open source community active in the fields of geospatial planning, local energy access solutions, and open science education. The comparison between advances of open source software and open source hardware is only partially legitimate as hardware is not a purely digital product that can be copied with negligible marginal cost. This material cost however, is rapidly reducing in comparison with the costs for development and maintenance, which is common between software and hardware. Future work on the role of open source in entrepreneurial ecosystems will require a closer look at legal obstacles and the regulatory frameworks that may be lagging behind the rapid transition to open source solutions. Open source hardware development also requires a redefinition of productivity and market valuation as the actual value of such open source products and services are mostly based on saving costs rather than supply and demand dynamics and price allocation by rational agents in a competitive market.

## Acknowledgments

This work has been supported by the FAIR Battery grant from the Centre for Unusual Collaborations at the EWUU Alliance. The authors thank the Open Source Community Africa for discussions and their input. VB acknowledges funding from the 10.13039/100004428Charles Stewart Mott Foundation.

## Author contributions

S.F., V.B., and D.M. conceived the first draft. S.F. wrote the draft manuscript. All authors contributed to the discussions and conclusions.

## Declaration of interests

V.B. is the CEO of the EnAccess Foundation. D.M. is the project lead of the Energy Access Explorer at the World Resource Institute.

## Declaration of generative AI and AI-assisted technologies

During the preparation of this work, the authors used Scopus AI for screening the background literature. After using this tool or service, the authors reviewed and edited the content as needed and take full responsibility for the content of the publication.
